# Cutaneous manifestations of infective endocarditis as presenting signs of left atrial myxoma in a patient with acute ischemic stroke: A case report

**DOI:** 10.1097/MD.0000000000039088

**Published:** 2024-09-06

**Authors:** Ying-Chi Shen, Kai-Chun Chang, Jen-Jen Su

**Affiliations:** a Department of Neurology, National Taiwan University Hospital, Taipei, Taiwan; b Department of Internal Medicine, Cardiovascular Center and Division of Cardiology, National Taiwan University Hospital, Taipei, Taiwan.

**Keywords:** cardioembolism, cutaneous embolization, infective endocarditis, myxoma, stroke

## Abstract

**Rationale:**

Approximately one-fifth ischemic stroke are attributed to cardioembolism. Patients with cardioembolic stroke often develop a more severe disability and a higher risk of stroke recurrence. Cardiac myxoma, although uncommon, can serve as a potentially curable cause of acute embolic strokes.

**Patient concerns:**

A 55-year-old male patient presented to the emergency department with acute vertigo and unsteady gait, accompanied by left upper limb numbness. Concurrently, purple-like lesions on the left hand were noticed.

**Diagnoses:**

Brain magnetic resonance imaging showed multiple infarctions in the posterior circulation. Additionally, skin examination showed Janeway lesions, Osler nodes and splinter hemorrhages. There was no evidence of systemic infection. Subsequently, transthoracic echocardiogram revealed a left atrial myxoma.

**Intervention:**

Early surgical resection of cardiac myxoma was performed.

**Outcomes:**

The patient recovered well from the surgery. No recurrent embolic event was reported at 3-month postoperatively.

**Lessons:**

Clinicians should be vigilant for skin manifestations of cardiac embolism. In patients with acute ischemic strokes, the presence of cutaneous embolic phenomena could serve as a warning sign of cardioembolism.

## 1. Introduction

Cardioembolic stroke accounts for about one-fifth of acute ischemic strokes.^[1]^ The risk of early stroke recurrence and mortality are relatively high following a cardioembolic stroke.^[1]^ Therefore, comprehensive investigations for cardioembolism are warranted if the clinical presentation highly suggests embolic strokes. Cardiac myxoma (CM) is an infrequent yet treatable etiology of cardioembolic stroke.^[2]^ Patients with CM-related ischemic stroke can initially present with a variety of clinical manifestations. However, they often develop a triad of obstructive cardiac symptoms, systemic embolization, and constitutional symptoms.^[3]^ Approximately 16% of patients with CM presented with peripheral systemic embolization as an initial symptom, which can also occur at any time in one-third of patients.^[3]^ Although early diagnosis of CM can be challenging, some literature reported that transient cutaneous manifestations may serve as a diagnostic clue for development of catastrophic thromboembolic events.^[4,5]^ In this report, we present a rare case of a patient with multiple embolic strokes in the posterior circulation, who exhibited skin lesions mimicking infective endocarditis (IE), and was later diagnosed with left atrial myxoma.

## 2. Case report

A 55-year-old male patient, a heavy smoker with 30 pack-year smoking history and no known systemic disease, presented to the emergency department with acute-onset vertigo accompanied by numbness in his left upper limb. Previously, he experienced 2 episodes of nonvertiginous dizziness that resolved after sleeping, occurring within the past 6 months. On the day of presentation, while driving, he developed acute-onset vertigo again, along with nausea, slurred speech, and left upper limb numbness with tingling sensations in his left distal fingers. Additionally, he developed an unsteady gait when he attempted to exit the car. He denied having a headache, blurred vision, diplopia, tinnitus, dysphagia, or dyspnea. The patient was brought to the emergency department by his family within 1 hour of symptoms onset.

Upon arrival at the hospital, the patient’s vital signs were stable, with a blood pressure of 116/73 mmHg, a temperature of 35.9 °C, a pulse rate of 84/min, and a respiratory rate of 20/min. The initial neurological examination revealed gaze-evoked nystagmus, mild weakness of the left upper limb (Medical Research Council Scale for muscle strength of the left upper extremities, grade 4+), and hypesthesia and dysmetria of the left extremities. Based on these clinical findings, an acute stroke was suspected. The National Institutes of Health Stroke Scale (NIHSS) score was 3, with 2 points for ataxia and 1 for sensory loss. Non-contrast brain computed tomography (CT) showed no intracranial hemorrhage or mass lesions. The patient did not receive intravenous thrombolysis due to the minor, nondisabling stroke; instead, he was started on dual antiplatelet therapy with aspirin and clopidogrel for secondary stroke prevention.

**Figure 1.**
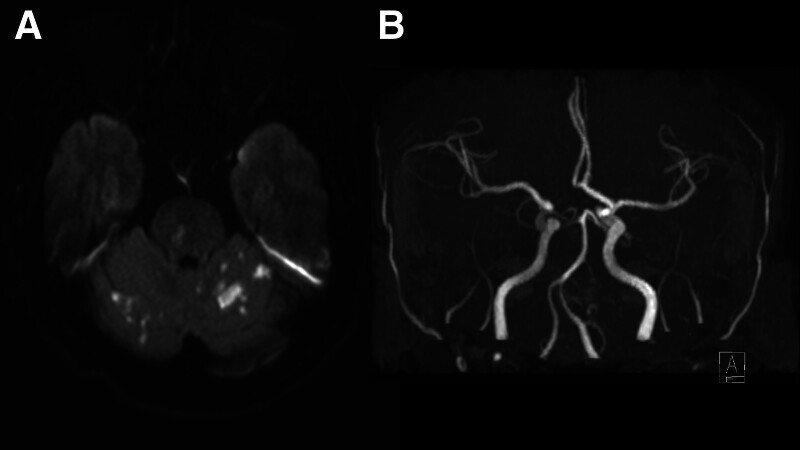
Diffusion-weighted MR imaging revealing multiple recent infarctions in the posterior circulation involving bilateral cerebellum and pons (A); MR angiography showing no evidence of stenosis of the major intracranial arteries (B). MR = magnetic resonance.

**Figure 2.**
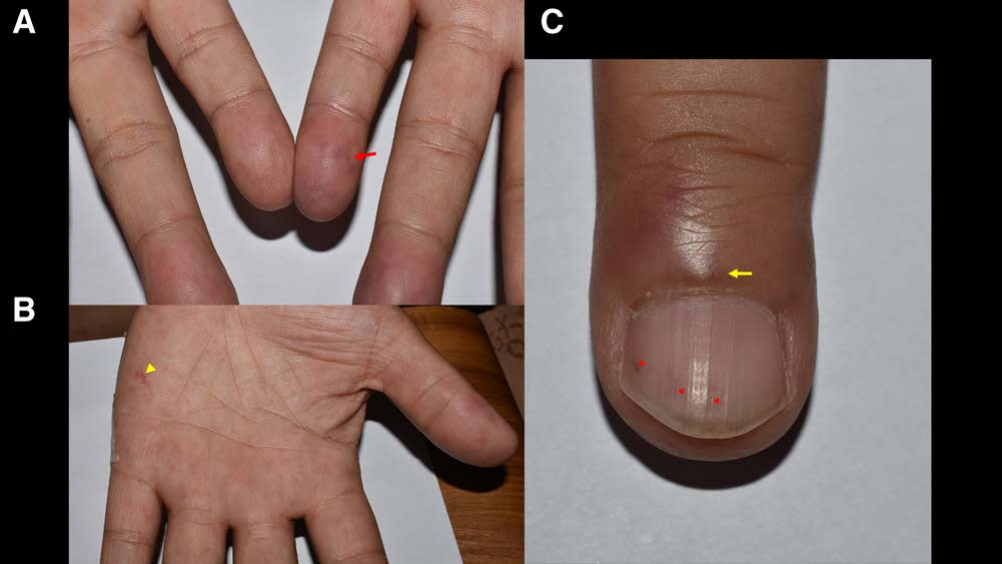
The cutaneous findings of our patient included (A) cyanotic changes over the left fingertips (red arrow), (B) a few erythematous to violaceous, non-tender, macules on the left palm indicative of Janeway lesions (yellow arrowhead), and (C) an erythematous, tender nodule on the left third finger suggestive of Osler nodes (yellow arrow), along with splinter hemorrhages on the left fingernails (red arrow head).

The patient was admitted for a comprehensive investigation of acute stroke. Subsequent brain magnetic resonance (MR) imaging revealed multiple recent infarctions in the posterior circulation, involving the bilateral cerebellum and pons (Fig. 1A). MR angiography showed no stenosis of the major intracranial arteries (Fig. 1B). Laboratory workups including tests for vascular risk factors, coagulopathy, and vasculitis screening, were unremarkable. The 12-lead electrocardiography (EKG) showed a normal sinus rhythm. On the day of admission, the patient reported unusual purple-like lesions on the left hand, and a constant feeling of coldness in both hands. However, there were no diminished or absent pulses in the distal extremities. Doppler ultrasound of the upper extremities revealed patent arteries of the bilateral upper limbs, making the diagnosis of peripheral arterial occlusion disease unlikely. Notably, a comprehensive skin examination revealed several cutaneous findings: cyanotic changes over the left fingertips (Fig. 2A), a few erythematous to violaceous, non-tender, macules on the left palm indicative of Janeway lesions (Fig. 2B), an erythematous, tender nodule on the left third finger suggestive of Osler nodes, and splinter hemorrhages on the left fingernails (Fig. 2C). These findings led to a high suspicion of cutaneous thromboembolism. Given the presence of multiple embolic strokes and cutaneous embolic phenomena, cardioembolism was considered the most likely etiology of the stroke.

**Figure 3.**
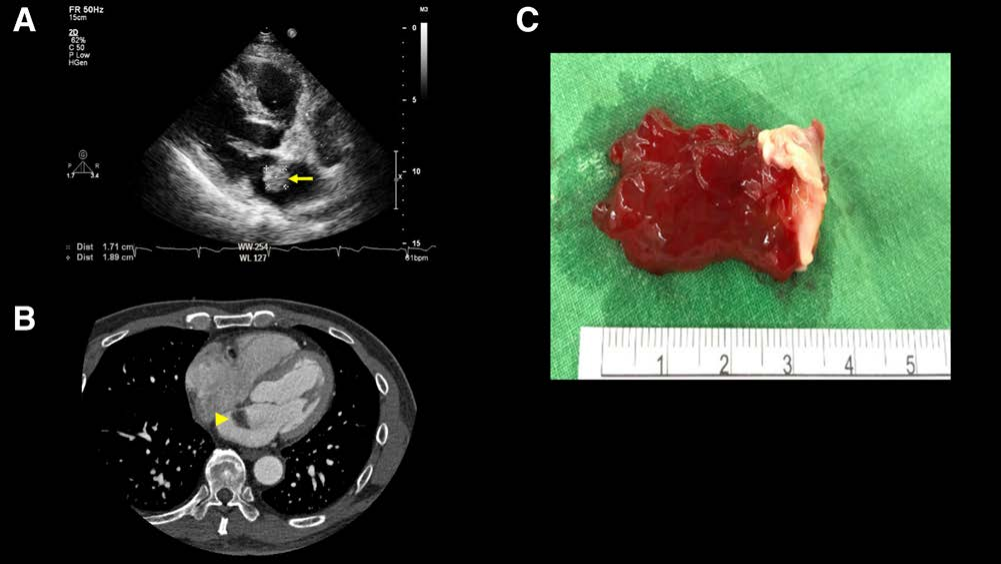
(A) Transthoracic echocardiography showing a mass attached to the interatrial septum and protruding into the left atrium (yellow arrow). (B) EKG-gated cardiac CT revealing a 2.77-cm floating fat-containing nonenhancing lesion (yellow arrowhead) adhering to the interatrial septum and protruding into the left atrium. (C) The picture showing gross specimen of the excised tumor, measuring 3.2 × 2.0 × 1.2 cm in size.

During hospitalization, the patient neither develop a fever nor exhibited heart murmurs. Due to the specific cutaneous lesions, 2 sets of blood cultures were collected for suspected IE; however, they showed no growth after 5 days. A transthoracic echocardiography identified a 1.71 × 1.89 cm mass in the left atrium, highly suggestive of a myxoma (Fig. 3A). For higher spatial and temporal resolution, an EKG-gated cardiac CT was performed, revealing a fat-containing, nonenhancing, floating lesion adhered to the interatrial septum, protruding into the left atrium (Fig. 3B). After consultation with a cardiovascular surgeon, early surgery was recommended due to the high risk of recurrent stroke. Subsequently, antiplatelet agents were discontinued after confirming the diagnosis of CM.

On the tenth day after the onset of stroke, the patient underwent left atrial tumor excision. The final pathological result confirmed a left atrial myxoma. (Fig. 3C). The postoperative period was uneventful and at the 3-month follow-up visit, the patient reported no neurological deficits, and his skin lesions had resolved.

## 3. Discussion

We report a case of left atrial myxoma that initially presented as an acute embolic stroke, accompanied by Osler node and Janeway lesions. This case highlights the importance of a thorough head-to-toe skin examination to detect any signs of cutaneous micro-emboli once an embolic stroke is diagnosed. Such cutaneous embolization could serve as early warning indicators for CM. Timely diagnosis and resection of myxoma are critical to prevent potential catastrophic cerebral or systemic embolization.

CMs are the most common primary cardiac tumors in adults, accounting for approximately 80% of all primary neoplasms of heart.^[6]^ Although histologically benign, CMs can cause severe embolic complications.^[6]^ About 80% of CMs are found in the left atrium, often leading to clinical manifestations such as mitral valve obstruction, systemic embolization and constitutional symptoms.^[6]^ Neurological complications are observed in 10% to 15% of patients with CMs, with cerebral infarction being the most frequent neurological manifestations.^[3,6]^ CM could be a great imitator with its variable clinical spectrum, ranging from being entirely asymptomatic to presenting with life-threatening embolic events or even cardiac death.^[7]^ Research indicated that the mobility and irregular surface of the CM, rather than its size, were more likely to be associated with an increased risk of embolism.^[3,8]^ Therefore, systemic embolization may occur before a CM is large enough to be detected on cardiac imaging.

Cutaneous manifestations may serve as sentinel signs for CM.^[9]^ The cutaneous manifestations observed in our patient, such as Osler nodes and Janeway lesions, share similar features with those seen in patients with IE.^[10,11]^ Janeway lesions are described as painless erythematous macules or papules on the palms or soles, often seen in the acute stage of IE. Osler nodes, in contrast, appear as painful subcutaneous violaceous nodules in the glomus apparatus of the fingers and toes, commonly associated with subacute bacterial endocarditis.^[12]^ The pathomechansim differs for each: Janeway lesions are believed to result from septic microembolism, while Osler nodes are thought to arise from an immunological response to the infectious agent causing endocarditis.^[12]^ Nevertheless, both lesions can indicate systemic cutaneous embolization, necessitating urgent cardiac imaging to exclude cardioembolism. These cutaneous findings are not only exclusive to IE and can be found in a variety of conditions, such as nonbacterial thrombotic endocarditis, vasculitis, and chronic infectious disease.^[12]^ Other documented cutaneous embolism in patients with CM included transient and recurrent painful acral erythematous papules or macules, acral petechiae, digital cyanosis, splinter hemorrhages, livedo reticularis, ulcerative lesions and Raynaud phenomenon.^[5,13,14]^ One study revealed that skin manifestation may be linked to higher rate of extracardiac complications and a worse prognosis in patients with endocarditis.^[10]^

Our report broadens the clinical understanding of left atrial myxoma by presenting a rare case that features both acute posterior circulation embolic stroke and cutaneous embolization. It also emphasizes the importance of a comprehensive cutaneous examination in patients diagnosed with multiple embolic strokes.

## 4. Conclusion

Clinicians should be vigilant for skin manifestations of cardiac embolism. In patients with acute ischemic strokes, the presence of cutaneous embolic phenomena could serve as a warning sign of cardioembolism. Urgent cardioembolic investigations are warranted upon detecting cutaneous microembolism to facilitate the early diagnosis of CM.

## Acknowledgments

We would like to thank the patient and his sister for their participation and consent for publication.

## Author contributions

**Conceptualization:** Ying-Chi Shen.

**Supervision:** Jen-Jen Su.

**Validation:** Ying-Chi Shen, Jen-Jen Su.

**Visualization:** Ying-Chi Shen, Kai-Chun Chang.

**Writing – original draft:** Ying-Chi Shen.

**Writing – review & editing:** Jen-Jen Su, Kai-Chun Chang.
